# Changing Conspiracy Beliefs through Rationality and Ridiculing

**DOI:** 10.3389/fpsyg.2016.01525

**Published:** 2016-10-13

**Authors:** Gábor Orosz, Péter Krekó, Benedek Paskuj, István Tóth-Király, Beáta Bőthe, Christine Roland-Lévy

**Affiliations:** ^1^Department of Social Psychology, Institute of Psychology, Eötvös Loránd UniversityBudapest, Hungary; ^2^Research Centre for Natural Sciences, Institute of Cognitive Neuroscience and Psychology, Hungarian Academy of SciencesBudapest, Hungary; ^3^Central Eurasian Studies Department, Indiana UniversityBloomington, IN, USA; ^4^Department of Psychology, University College LondonLondon, UK; ^5^Doctoral School of Psychology, Eötvös Loránd UniversityBudapest, Hungary; ^6^C2S, Department of Psychology, University of Reims Champagne-ArdenneRheims, France

**Keywords:** conspiracy theory, belief, rationality, ridiculing, empathy

## Abstract

Conspiracy theory (CT) beliefs can be harmful. How is it possible to reduce them effectively? Three reduction strategies were tested in an online experiment using general and well-known CT beliefs on a comprehensive randomly assigned Hungarian sample (*N* = 813): exposing rational counter CT arguments, ridiculing those who hold CT beliefs, and empathizing with the targets of CT beliefs. Several relevant individual differences were measured. Rational and ridiculing arguments were effective in reducing CT, whereas empathizing with the targets of CTs had no effect. Individual differences played no role in CT reduction, but the perceived intelligence and competence of the individual who conveyed the CT belief-reduction information contributed to the success of the CT belief reduction. Rational arguments targeting the link between the object of belief and its characteristics appear to be an effective tool in fighting conspiracy theory beliefs.

## Introduction

The level of beliefs in conspiracy narratives differs among people. Conspiracy narratives claim that powerful people or organizations cooperate in secret, to achieve sullen objectives by deceiving the public (Abalakina-Paap and Stephan, 1999; Wood et al., 2012; Wood and Douglas, 2013). According to 160 million Americans, there was a conspiracy behind JFK's murder; today, 110 million people believe that global warming is a hoax; 18 million believe that Bin Laden is still alive; and 12 and a half million believe that non-human beings, the so called Lizard people (they are the reptilian elite controlling the world since ancient times) control politics (Williams, 2013). Conspiracy theories are relevant for social interaction and democracy as they can induce anger, lead to low political participation, and to learned helplessness. While some studies have shown that inducing conspiracy theories is easy (Butler et al., 1995), to date, only a few researchers have attempted to examine the possibility of reducing beliefs in conspiracy theories (Banas and Miller, 2013); there is no accumulated knowledge about effective methods of CT reduction. The present study intends to fill this gap.

Despite theoretical diversity (see Krekó, 2015, for an overview), a few characteristics of CTs appear to be consensual. First, CTs are associated with a mechanistic worldview covering the beliefs that (a) nothing happens by chance; (b) nothing is what it seems; (c) everything interconnects with everything (Barkun, 2003). Second, CTs are organically connected to each other and are likely to be integrated in a lay conspiracy meta-theory of society (Goertzel, 1994). Although CTs are organically connected, they can be categorized on the basis of their scope: (a) they can explain concrete events (e.g., JFK); (b) systematic CTs cover a group's attempt for hegemony; (c) super CTs refer to a complex conspiracy worldview, including several seemingly independent theories (Barkun, 2003). Third, CT's are characterized by a bipolar, black, and white logic, which divides society into good and evil (Moscovici, 1987; Berlet, 2009). Fourth, CTs resist rational arguments, because only the pieces of information, which are consistent with them, are processed, the incompatible ones being rejected (Bartlett and Miller, 2010). The present study intends to challenge this latter characteristic of CTs with an experimental study.

According to previous studies, individual differences appear regarding beliefs in CTs. These individual differences can be categorized into personality and attitudinal dimensions. Among personality variables, belief in CTs is negatively related to self-esteem (Abalakina-Paap and Stephan, 1999; Swami et al., 2011), to agreeableness (Swami et al., 2011; Bruder et al., 2013), and to conscientiousness among Big Five traits (Brotherton et al., 2013). Based on attitudinal variables, beliefs in CTs are positively related to feelings of powerlessness (Abalakina-Paap and Stephan, 1999; Bruder et al., 2013), to perceived lack of control (Hamsher et al., 1968; Whitson and Galinsky, 2008), to mistrust of other people (Goertzel, 1994; Abalakina-Paap and Stephan, 1999; Brotherton et al., 2013), and authorities (Swami et al., 2010), anomie (Goertzel, 1994; Abalakina-Paap and Stephan, 1999; Brotherton et al., 2013; Bruder et al., 2013), and authoritarianism (Goertzel, 1994; Abalakina-Paap and Stephan, 1999; Swami et al., 2011; Bruder et al., 2013). The only demographic variable positively related to beliefs in CTs was minority status (Goertzel, 1994; Crocker et al., 1999). Nevertheless, these individual differences were only weakly related to beliefs in CTs.

If conspiracy theories are associated with malevolent effects, the question arises: How is it possible to change conspiracy beliefs? According to Fishbein and Ajzen (1975), beliefs can be defined as one's subjective probability judgments related to a given aspect of the perceived world. Beliefs contribute to the understanding of oneself and depend on the person's environment. CTs are mainly inferential beliefs because they go beyond the observable events and are derived from external sources. These sources provide certain pieces of information; thus they can be interpreted as informational beliefs. CTs, as other beliefs, have an object (Jews, European Union, global financial system, or bankers, etc.), which is related to a given attribute (exploitative, hidden, and manipulative, etc.). The link between the object and the attribute exists on a certain level, based on subjective probability judgment.

Based on Fishbein and Ajzen's conceptualization (Fishbein and Ajzen, 1975), belief change refers to the modification of subjective probability judgments. Based on the theory of planned behavior^1^ (Ajzen, 1985, 1991), we assume that it is possible to change these probability judgments in three ways. First, it is possible to (a) change the link between the object and the attribute. Second, it is possible to (b) increase the distance between the self and those who hold a relationship between the object and the attribute. Third, it is possible to (c) manipulate the level of identification between the object and the person who holds a certain level of conspiracy belief toward the object. All three of these strategies can be anchored to pre-existing attitude change theoretical frameworks: the functional attitude theories (Katz, 1960), the elaboration likelihood model with its central and peripheral routes (Petty and Cacioppo, 1986), and the consistency theories (e.g., Abelson, 1968).

(a) In order to change the link between the object and the attribute, further logical pieces of information or logical steps can be provided, thus allowing us to elaborate on the logical structure which can result in a more complex link. For example, the object of the belief can be the banks, and the attribute can be exploitative. A low level of elaboration lacks arguments as to why this link exists, whereas a high level of elaboration includes many logically compatible and underpinned arguments. We hypothesize that pointing out the logical flaws of this link, providing an elaborated explanation without logical flaws could make one deconstruct the preexisting link and construct a new one between the object and the attribute. This should lead to belief changes. In the previous example, if we provide detailed information about how much money banks get from taxpayers' money, which is four times less than what they pay to the state in many different special taxes in any given period of time, the link becomes more elaborate in terms of complexity and coherence. We assume that this can lead, to some extent, to belief change. From the perspective of functional attitude theories (Katz, 1960), rational arguments affect the knowledge function by providing detailed information about the link between the object and the attribute of the belief. This strategy can provide a deeper sense of understanding and control over the particular conspiracy-related part of the external world. From the perspective of the elaboration likelihood model (Petty and Cacioppo, 1986), the rational strategy is related to the central route of persuasion in which an individual holding CT beliefs evaluates the pros and cons of the rational arguments and estimates the fit of these detailed arguments to the pre-existing value system. From the perspective of inconsistency theories (Abelson, 1968), belief rationalization in the three arguments present different forms of inconsistency. Regarding those who hold CT beliefs, showing data through rational arguments can create inconsistencies between the informational content of the previous beliefs and the new information regarding events or people related to the conspiracies. This inconsistency can be reduced by challenging the original conspiracy beliefs.

(b) The second possibility of conspiracy belief change involves increasing the distance between the self and those who hold a certain link between the object and the attribute. To achieve this, one can demonstrate that those people who hold such beliefs are characterized by negative traits or they are targeted as being ridiculous. As practically no one wants to be ridiculed by others, the ridiculing argument can be fueled by the ego-protective function (Katz, 1960). For example, if one shows that people who believe that Osama Bin Laden is still alive, while also believing that he was already dead before the US troops found him (see Wood et al., 2012), they cannot be taken seriously. Nevertheless, this is a part of CT beliefs and suggests that CT believers have difficulties with logical thinking. In this case, an individual may choose to increase the distance between the self and those who hold a certain link between the object (mass media/Osama Bin Laden) and the attribute (misleading/dead). If one considers the elaboration likelihood model (Petty and Cacioppo, 1986), the ridiculing method belongs to the peripheral routes. It is affected by less deliberate processes either through building more positive emotional bonds to the object of the beliefs or by increasing the gap between the self and those who hold such beliefs as a result of ridiculing arguments. Furthermore, we assume that those individuals who have had prior conspiracy beliefs will experience a certain level of cognitive dissonance (Festinger, 1962; Aronson, 1969). The source of dissonance is the opposition between being a logical person and the irrationality of CT believers. Ridiculing arguments can create inconsistency as a result of social identity issues, that is, that the individual does not want to belong to a ridiculous group (Abelson, 1968). This dissonance can be reduced through changing the CT beliefs; however, it also needs to question (a) the assumption that CT believers are irrational, as well as (b) the source of this information. As far as we know, this self-distancing aspect of belief change has been relatively poorly investigated in social psychology. However, there are some successful belief change examples from recent history. One of these is the reduction of the popularity of the Ku-Klux-Klan (KKK): in the 1940's Stetson Kennedy exposed many of the KKK's secret rituals (handshakes, passwords, and other ludicrous behaviors) in a ridiculous manner. The consequences were immediate, after 2 weeks, the recruitment of KKK plummeted to zero. People did not want to join an organization because the formerly terrifying shadow organization of white pride was now regarded as laughable. Therefore, we presume that, in this case, the belief change occurred as a result of distancing one's self from KKK members because of their pathetic and ridiculous practices.

(c) We suppose that the third form of conspiracy belief change relates to the identification with the object of the belief. Therefore, in this case, the primary goal is not to change the link between the object and the attribute, but to focus on the reduction of the distance between the self and the object of the CT. For example, CTs concerning objects (e.g., Jews) generally include negative attributes (e.g., secretly manipulative). If, as a result of a message (e.g., claiming that Christians faced similar conspiracy theories beforehand), people put ourselves in the position of Jews, they can empathize more easily with them, which can lead to more positive attitudes toward them. These empathizing arguments can be related to value expressive functions in terms of presenting an image that is in line with a positive and caring self-concept (Katz, 1960). The empathizing arguements do not necessarily directly influence the link between the object (e.g., Jews) and the negative attribute (e.g., secretly manipulative), but they can provide an alternative evaluative dimension of the object, which can indirectly weaken the original link. From the perspective of the elaboration likelihood model (Petty and Cacioppo, 1986), contrary to the rational arguments, the empathic ones are related to the peripheral route. On the basis of the consistency theories (Abelson, 1968), empathy creates inconsistency on the level of the target group of the beliefs who have positive attributes besides the negative ones that a conspiracy believer mostly associates with them. Finally, since objects of CTs are generally prejudiced groups (e.g., Jews, speculators, bankers, Chinese, or Lizard people), on the basis of prejudice reduction research, empathy toward these groups can reduce the negative prejudices which may lead to the CT belief change (e.g., Pettigrew and Tropp, 2008; Berger et al., 2016).

The goal of the present study was to experimentally examine the above-described three strategies of CT belief change in an online setting on comprehensive samples. We expected that all three strategies would effectively reduce CT beliefs.

## Materials and methods

### Participants

This research employed a nationally representative probability sample of 813 Hungarians, selected randomly from an Internet-enabled panel, including 20,000 members, with the help of the Solid Data Ltd., in October–November 2014. For the preparation of the sample, a multiple-step, proportionally stratified, probabilistic sampling method was employed. Members of this panel used the Internet at least once a week. The demographic characteristics of the panel are permanently filtered. More specifically, individuals were removed from the panel if they responded too quickly (i.e., without paying attention to their response) and/or had fake (or not used) e-mail addresses. The sample was nationally representative in terms of gender, age, level of education, and location of residence. The study was conducted in accordance with the Declaration of Helsinki and with the approval of the Institutional Review Board of the Eötvös Loránd University. Before starting the questionnaire, participants received information about the study in terms of examining beliefs about how societies work. Subsequently, participants read and approved the informed consent.

Eight hundred and thirteen (813) individuals (50.8% female) participated in the present online experiment, between the ages of 18 and 75 (*M* = 45.7, *SD* = 15.04). Participants were randomly assigned to the conditions, taking into account the representativeness (age, gender, level of education, place of residence). In the experimental conditions, participants listened to the CTs instead of reading them. Only the participants, who listened to the CTs and the audio recordings of the experimental manipulations until the end, were selected. As a result, 104 participants were excluded. The final sample consisted of 709 participants (51.1% female), the average age was 46.43 (*SD* = 14.74), the age range being 18–75 years. Regarding the places of residence, 209 (29.5%) respondents lived in the capital, 217 (30.6%) lived in county capitals, 138 (19.5%) lived in other towns, and 145 (20.5%) lived in villages. Regarding the levels of education, 120 (16.9%) respondents had only received primary school education, 212 (29.9%) had a secondary level education, 377 (53.2%) had a higher level education, which implies that the sample was better educated than the offline population.

### Procedure

In the present study, participants were recruited via Internet. After agreeing with the informed consent form, participants listened to the first audio recording (for the transcript, see Appendix 1 in Supplementary Material). This is a 4:30 min recording that presented a conspiracy super theory including the victimization of Hungary by the financial imperium, the hidden control of Jews over the world, the EU as a non-functional oppressive power, and the bankers who exploit the Hungarian financial system. The text provided vivid, but confusing details about the mechanisms that “actually” shape the fate of Hungary and the world. This super CT met the above mentioned characteristics of CTs in terms of nothing happens by chance, nothing is what it seems, everything is interconnected with everything, and the world is divided into good and evil.

Having listened to the recording, participants expressed their acceptance concerning eight questions on the four main topics (victimization of Hungary, EU, power of the Jews, bankers). Then, they were asked about their general acceptance of the listened CT. The final two questions referred to the perceived competence and intelligence of the speaker who reported the CT. After this questionnaire, participants were randomly assigned to one of the four conditions. They listened to another speech with, either rational (3:36 min), ridiculing (3:28 min), or empathetic (2:54 min) arguments against CTs. In the control condition (3:15 min), they listened to a weather forecast. The transcripts of each condition can be seen in Appendix 2 in Supplementary Material.

In the rational condition, the text tackled the claims made in the first recording in a logically plausible manner, using numbers to support the objections, and pointing out the discrepancy between high influence and concealment. This speech pointed out the logical flaws of the first speech and corrected it with in-depth arguments regarding the link between the beliefs' objects and attributes. The goal of this condition was to emphasize the logical inconsistencies and to create a more complex and coherent relationship between the objects of the belief and the attributes.

In the ridiculing condition, the script addressed the same logical flaws, but reasoned against them differently: instead of focusing on certain details, it derided the logical inconsistencies and concentrated on those who believe in the CTs, picturing them as evidently ridiculous (e.g., mentioning the believers of Lizard Men). This text intended to increase the distance between the respondents' self and those who believe in CTs.

The empathetic condition contested the original text's claim in a different manner: instead of focusing on content or those who believe in the content, it placed the objects of the CTs in the center, and compassionately called attention to the dangers of demonizing and scapegoating, while also pointing out the human character of the CT objects (i.e., Jews face similar conspiracy theories and persecution nowadays that the Early Christians faced). This condition intended to reduce the distance between the respondent and the objects of CTs and to raise empathy toward these groups.

In the control condition participants listened to a simple weather forecast which was not related to the content of the CT. After the recording, respondents answered the same 11 questions.

### Measures

#### Conspiracy assessment tool (CAT)

An eight item scale was created specifically for the present experiment to measure the individual's attitudes toward conspiracies, assessing beliefs regarding conspiracies related to four aspects: (1) Hungary as a victim of conspiracy; (2) Jews as the leaders of the world; (3) the European Union is a parasitic formation without any function; and (4) the bankers as the leaders of the world. These specific topics were related to the CT audio they listened to previously. More precisely, they referred to the key elements of the text regarding these four topics. Respondents used an 11-point scale to indicate their level of agreement (0, Strongly Disagree; 10, Strongly Agree). This scale showed high levels of reliability with a Cronbach alpha value of 0.95 for the pre-manipulation and 0.96 for the post-manipulation.

In addition to the eight CAT items, one item measured the listener's level of agreement with the audio excerpts, using the response options of the previous eight items. Finally, two additional items were created: the first assessed the competence of the speaker on an 11-point scale (0, Not competent at all; 10, Completely competent), while the second item assessed the intelligence of the speaker on a 5-point scale (1, Far below average; 5, Far above average).

#### Conspiracy mentality questionnaire (CMQ)

This 5-item measurement was developed by Bruder et al. (2013) and was translated, by following Beaton et al.'s (2000) protocol. Furthermore, six additional items reflecting on the Hungarian societal context were added. The CMQ measures the tendency to engage in conspiracy-related ideations (Cronbach α: 0.80). Respondents used a 7-point scale for answering (1, Not true at all; 7, Completely true).

#### Balanced inventory of desirable responding (BIDR)

The 40-item BIDR (Paulhus, 1991) was administered to assess social desirability. This measure contains two subscales (self-deceptive positivity and image management), with 20 items belonging to each. In the present study, internal consistency indices were acceptable (Cronbach α_self−deceptive enhancement_: 0.71; Cronbach α_impression management_: 0.55). Respondents used a 7-point scale for answering (1, Not true at all; 7, Completely true).

#### Big five (BFI)

Personality-related dimensions were assessed by the Big Five thInventory (John and Srivastava, 1999). The BFI is a 45-item scale that measures the personality of the respondent according to five dimensions: extraversion (Cronbach α: 0.69), agreeableness (Cronbach α: 0.62), conscientiousness (Cronbach α: 0.62), emotional stability (Cronbach α: 0.74) and openness (Cronbach α: 0.84). In this study, a shorter, valid version was used (Farkas and Orosz, 2013) that contained 15 items; respondents used a 5-point scale to indicate their level of agreement (1, Strongly disagree; 5, Strongly agree).

#### Demographic variables

Participants were asked to indicate the following demographic variables: age, gender (1, female; 2, male), place of residence (1, capital; 2, county town; 3, town; 4, village), and level of education (1, primary level education; 2, secondary level education; 3, higher level education).

## Results

Descriptive statistics of the above-mentioned questionnaires with ranges, summed scores, standard deviations, and intercorrelations can be seen in Table 1 (see the Dataset here). Individual differences in terms of Big Five traits and BIDR dimensions were either unrelated to or very weakly related to CAT scores. Only Openness and BIDR-SDE were negatively and weakly related to CAT scores. Finally, CMQ and CAT were highly associated with each other.

**Table 1:** **Descriptive statistics and correlations of the included questionnaires (*N* = 709)**.

**Scales**	**Range**	**Mean**	***SD***	**1**	**2**	**3**	**4**	**5**	**6**	**7**	**8**	**9**
1. Conspiracy Assessment Tool—pre	8–88	45.15	25.88	–								
2. Conspiracy Assessment Tool—post	8–88	43.28	26.11	0.94^**^	–							
3. Conspiracy Mentality Questionnaire	11–77	51.08	11.21	0.60^**^	0.61^**^	–						
4. Self-deception Enhancement factor of BIDR	20–140	92.13	12.46	−0.09^*^	−0.07	0.02	–					
5. Impression Management factor of BIDR	20–140	82.36	11.44	−0.06	−0.04	−0.01	0.29^**^	–				
6. Extraversion	3–15	10.62	2.67	−0.01	0.00	0.04	0.20^**^	0.04	–			
7. Agreeableness	3–15	11.42	2.13	0.07	0.08^*^	0.11^**^	0.20^**^	0.20^**^	0.25^**^	–		
8. Conscientiousness	3–15	10.34	2.53	−0.01	−0.03	0.04	0.33^**^	0.34^**^	0.09^*^	0.14^**^	–	
9. Emotional stability	3–15	9.93	2.73	−0.05	−0.04	0.01	0.43^**^	0.12^**^	0.11^**^	0.32^**^	0.14^**^	–
10. Openness	3–15	11.35	2.92	−0.13^**^	−0.13^**^	−0.05	0.17^**^	0.16^**^	0.16^**^	0.22^**^	0.11^**^	0.11^**^

* p < 0.05;

** *p < 0.01*.

### Measuring the effectiveness of the audio excerpts

No significant differences were found between the baseline (pre-test) measures considering the four conditions (all *p* > 0.208). The CONDITION ^*^ TIME ANOVA predicting the change in the extent of believing in conspiracy theories revealed significant main effects of TIME, *F*_(1, 705)_ = 32.49, *p* < 0.001, η_*p*_^2^ = 0.04; but not of CONDITION, *F*_(1, 705)_ = 1.38, *p* = 0.249, η_*p*_^2^ = 0.01. The interaction of CONDITION ^*^ TIME was significant, *F*_(3, 705)_ = 6.13, *p* < 0.001, η_*p*_^2^ = 0.03. CAT scores did not significantly differ between the groups at baseline (lowest *p* = 0.21).

**Figure 1:**
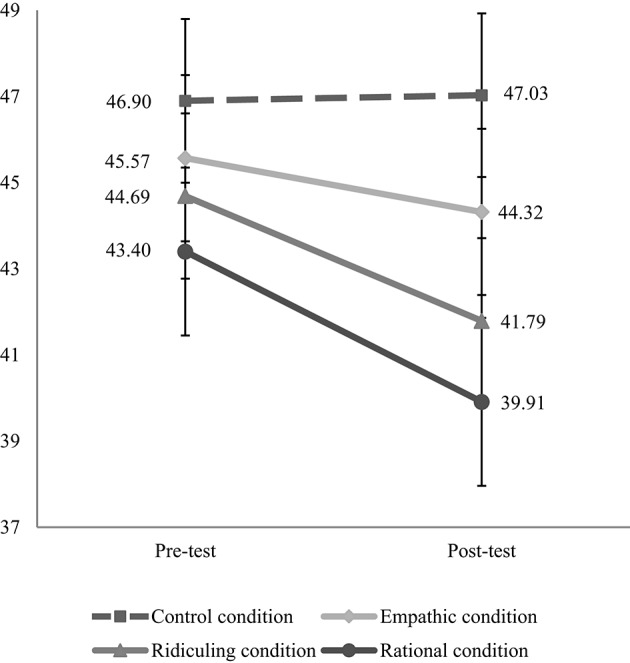
**Conspiracy Assessment Tool points before and after listening to audio excerpt related to conspiracy theory reduction in each condition**.

Paired-samples *T*-tests were conducted in order to investigate the change in pre-test and post-test CAT scores over time. CAT scores decreased significantly from pre-test to post-test among participants who participated in the rational condition, *t*_(171)_ = 4.32, *p* < 0.001, Cohen's *d* = 0.13 (for means, see Figure 1). Also, CAT scores decreased significantly from pre-test to post-test among participants who participated in the ridiculing condition, *t*_(177)_ = 5.46, *p* < 0.001, Cohen's *d* = 0.11. However, no significant decrease was found from pre-test to post-test in the other two conditions: empathetic, *t*_(185)_ = 1.71, *p* = 0.090, Cohen's *d* = 0.05, control, *t*_(172)_ = −0.28, *p* = 0.782, Cohen's *d* = 0.01. Compared to the post-test scores of the control condition, the rational condition had significantly lower post-test scores, *t*_(343)_ = 2.55, *p* = 0.011, Cohen's *d* = 0.27, the ridiculing condition tended to have lower scores, *t*_(349)_ = 1.90, *p* = 0.059, Cohen's *d* = 0.20, and the empathetic condition's mean was not different, *t*_(357)_ = 0.97, *p* = 0.333, Cohen's *d* = 0.10.

These results suggest that the manipulation in the rational and the ridiculing condition were successful. However, in the empathetic condition the extent of believing in conspiracy theories did not significantly decrease.

### Measuring the effect of social desirability on the manipulation

The CONDITION ^*^ TIME ANCOVA controlling for BIDR-SE predicting the change in the extent of believing in conspiracy theories controlling for the self-deceptive enhancement factor of social desirability revealed significant main effect of TIME, *F*_(1, 704)_ = 7.25, *p* = 0.007, η_*p*_^2^ = 0.01; and of BIDR-SDE *F*_(1, 704)_ = 4.53, *p* = 0.034, η_*p*_^2^ = 0.01; but did not of CONDITION, *F*_(3, 704)_ = 1.34, *p* = 0.262, η_*p*_^2^ = 0.01. The interaction of CONDITION ^*^ TIME remained significant, *F*_(3, 704)_ = 6.32, *p* < 0.001, η_*p*_^2^ = 0.03, but the interaction of TIME ^*^ BIDR-SDE was only marginally significant, *F*_(1, 704)_ = 3.79, *p* = 0.052, η_*p*_^2^ = 0.01. These results suggest that self-deceptive enhancement had a direct effect on CAT scores, but it had no significant effect on the interaction.

The CONDITION ^*^ TIME ANCOVA controlling for BIDR-IM predicting the change in the extent of believing in conspiracy theories controlling for the impression management factor of social desirability revealed significant main effect of TIME, *F*_(1, 704)_ = 6.30, *p* = 0.012, η_*p*_^2^ = 0.01; but did not of BIDR-IM *F*_(1, 704)_ = 1.53, *p* = 0.216, η_*p*_^2^ = 0.00; and did not of CONDITION, *F*_(3, 704)_ = 1.32, *p* = 0.268, η_*p*_^2^ = 0.01. The interaction of CONDITION ^*^ TIME remained significant, *F*_(3, 704)_ = 6.31, *p* < 0.001, η_*p*_^2^ = 0.03, but the interaction of TIME ^*^ BIDR-IM was only marginally significant, *F*_(1, 704)_ = 3.04, *p* = 0.082, η_*p*_^2^ = 0.004. These results suggest that impression management did not have a direct effect on CAT scores, nor did it have an effect on the interaction.

### Measuring the effect of the perceived competency and intelligence of the speaker on the manipulation

The CONDITION ^*^ TIME ANCOVA controlling for COMPETENCY predicting the change in the extent of believing in conspiracy theories controlling for the perceived competency of the speaker revealed only marginally significant main effect of TIME, *F*_(1, 704)_ = 2.84, *p* = 0.093, η_*p*_^2^ = 0.004; but did not of CONDITION *F*_(3, 704)_ = 1.63, *p* = 0.180, η_*p*_^2^ = 0.01; however, significant main effect was found of COMPETENCY, *F*_(1, 704)_ = 60.83, *p* < 0.001, η_*p*_^2^ = 0.08. The interaction of CONDITION ^*^ TIME was significant, *F*_(3, 704)_ = 6.11, *p* = 0.025, η_*p*_^2^ = 0.03, but the interaction of TIME ^*^ COMPETENCY was not significant, *F*_(1, 704)_ = 0.19, *p* = 0.666, η_*p*_^2^ = 0.0003. These results suggest that the perceived competency of the speaker had a direct effect on CAT scores, but it had no significant effect on the interaction.

The CONDITION ^*^ TIME ANCOVA controlling for INTELLIGENCE predicting the change in the extent of believing in conspiracy theories controlling for the perceived intelligence of the speaker revealed no significant main effect of TIME, *F*_(1, 704)_ = 1.13, *p* = 0.289, $\eta_{p}^{2}=0.002$; of CONDITION *F*_(3, 704)_ = 1.26, *p* = 0.288, $\eta_{p}^{2}=0.01$; however, significant main effect was found of INTELLIGENCE, *F*_(1, 704)_ = 36.23, *p* < 0.001, η_*p*_^2^ = 0.05. The interaction of CONDITION ^*^ TIME was significant, *F*_(3, 704)_ = 6.13, *p* = 0.025, η_*p*_^2^ = 0.03, but the interaction of TIME ^*^ INTELLIGENCE was not significant, *F*_(1, 704)_ = 0.03, *p* = 0.868, η_*p*_^2^ = 0.00004. These results suggest that the perceived intelligence of the speaker had a direct effect on CAT scores, but it had no significant effect on the interaction.

### Measuring the effect of personality-related variables on the manipulation (ANCOVA)

Using Big Five traits as covariates in the CONDITION ^*^ TIME ANCOVAs did not show either the main effect or the interaction of personality traits, except for Openness which had a significant main effect, *F*_(1, 704)_ = 12.48, *p* < 0.001, η_*p*_^2^ = 0.02. These results suggest that none of the personality traits had a direct effect on CAT scores, and it had no significant effect on the interaction.

### Measuring the effect of level of engaging in conspiracy-related ideations on the manipulation

The CONDITION ^*^ TIME ANCOVA controlling for CMQ predicting the change in the extent of believing in conspiracy theories controlling for the level of engaging in conspiracy-related ideations revealed significant main effects of TIME, *F*_(1, 704)_ = 9.40, *p* = 0.002, η_*p*_^2^ = 0.01; and of CMQ *F*_(1, 704)_ = 859.87, *p* < 0.001, η_*p*_^2^ = 0.55; but did not of CONDITION, *F*_(3, 704)_ = 0.22, *p* = 0.885, η_*p*_^2^ = 0.001. The interaction of CONDITION ^*^ TIME remained significant, *F*_(3, 704)_ = 5.63, *p* = 0.001, η_*p*_^2^ = 0.02; however, the interaction of TIME ^*^ CMQ was not significant, *F*_(1, 704)_ = 3.58, *p* = 0.059, η_*p*_^2^ = 0.01. These results suggest that the level of engaging in conspiracy-related ideations had no significant effect on the results of the experiment.

## Discussion

Most of the existing studies regarding the nature of conspiracy theories are descriptive; moreover, experimental research exploring the possibility of CT belief change is very rare (Banas and Miller, 2013; Swami et al., 2014). In the present study, the immediate effects of three types of belief change strategies on conspiracy belief change were investigated on comprehensive samples. According to the results, the empathetic arguments were not very effective. However, ridiculing and rational arguments were effective in CT belief change.

Previously, it was assumed that CTs resisted rational arguments, because only those pieces of information that were consistent with them were processed, while the incompatible ones were rejected (Bartlett and Miller, 2010). The present results are challenging this idea. CTs are characterized by a bipolar, black and white logic, which divides the society into good and evil. The success of the rational argument can be justified by the elaborated processing of the logical link between the target and the attribute. It is also possible that rational arguments can reduce CT beliefs by challenging the black and white thinking (Moscovici, 1987; Berlet, 2009). In the case of the rational condition, the convincing message aimed to elaborate the link between the objects (Jews, European Union, global financial system or bankers) and the attributes (exploitative, hidden, and manipulative). By pointing out the logical flaws and inconsistencies of CTs, it aimed to create a more complex and coherent link between the objects and the attributes. These results are in line with Swami et al.'s (2014) findings, according to which analytic thinking reduces conspiracist ideation. Helping analytic thinking by providing detailed explanations can reinforce deliberate processing of information. Swami et al. (2014) used a more tacit form of analytic thinking induction; nevertheless, on the basis of the present results it might be similarly effective if the rational arguments were directly given to the people. In both cases, skepticism becomes stronger and individuals become less willing to endorse those logical flaws, which are inherent parts of CT beliefs. In the rational condition, participants were informed about the logical flaws of the CT beliefs, but this speech did not refer directly to the characteristics of CT believers. Therefore, the stimulus of the rational condition was less threatening to those participants who were CT believers than the ridiculing condition, which might also contribute to the higher effectiveness of these arguments. One more explanation for the results is that it was not the rational arguments themselves, but the “rationality heuristics” associated with the message (logically-looking argumentation, many facts and numbers) that caused the impact of this condition. An experimentum crucis examining the two options should be done in order to be able to decide the real cause of the impact.

In the ridiculing condition, the arguments focused on the deficiencies of CT believers' thinking. This belief change can be expected as a result of the emerging distance between the self and those who hold CT beliefs. Ridiculing arguments are threatening for those who hold strong conspiracy beliefs, but among those who do not have very strong CT beliefs it can lead to disidentification from the group of CT believers. The stronger the belief in conspiracy, the stronger cognitive dissonance can be expected, and the results can be apparent in attitudes and behavior. We only examined beliefs, and in this field, cognitive dissonance can be resolved in two main ways: CT beliefs can be reduced and the new information regarding CT believers can be rejected. Considering the full sample, this strategy reduced CT beliefs. However, when we examined the effect of this condition among those who held strong vs. relatively weak CT beliefs separately, we found no significant difference between the effects of rational arguments: it was effective in both groups. Further, examination is needed in order to identify why ridiculing strategy did not have an overwhelming effect.

Among the experimental manipulations, the empathetic condition was the least effective. Previous studies found that perspective taking can effectively reduce CT beliefs (van Prooijen and van Dijk, 2014). In the present study, strengthening empathy toward the object of the CT beliefs did not seem to reduce CT beliefs effectively. Several reasons why we did not find this strategy effective can be considered. First, empathy and perspective taking can be considered as distinct constructs (Davis, 1983). Second, unlike van Prooijen and van Dijk (2014), the present study used CT beliefs with topics close to the respondents' everyday life (Hungary, EU, bankers, Jews); and these were probably more embedded than stories about distant events as in van Prooijen and van Dijk's study. We suggest that conspiracy beliefs can be more easily changed when they have less prevalent anchors in the life of the studied group. Consequently, more deeply rooted CT beliefs are harder to change by enhancing empathy toward the CT's object; however, emphasizing perspective-taking can be more beneficial for this purpose.

On the basis of these results, two belief changing strategies appeared to be effective: the rational and the ridiculing ones. From the perspective of the different theoretical backgrounds, there are alternative explanations for these findings. On the basis of the functional attitude theories (Katz, 1960), if someone wishes to reduce CT beliefs, the informational and ego-defensive functions of attitudes toward CTs could be equally employed in communication strategy building. From the perspective of the elaboration likelihood model (Petty and Cacioppo, 1986), both central and peripheral paths can be equally effective, especially if the peripheral one is more closely related to ridiculing those who hold these beliefs than empathizing with the victims of CT beliefs. Finally, the consistency theories (e.g., Abelson, 1968), in order to reduce the acceptance of CT beliefs, appear to be effective to create inconsistency between being a logical person (as a specific part of positive self-concept) vs. the irrationality of CT believers and between the informational content of the previous beliefs vs. the new information regarding events or people related to conspiracies.

Thanks to the rich literature regarding the links between individual differences in terms of personality (Abalakina-Paap and Stephan, 1999; Whitson and Galinsky, 2008; Swami et al., 2011; Brotherton et al., 2013; Bruder et al., 2013) and values (Abalakina-Paap and Stephan, 1999; Swami et al., 2011; Brotherton et al., 2013; Bruder et al., 2013), we presumed that individual differences could affect, to some extent, the effectiveness of the three strategies. According to the results, this is not the case. Individual differences in terms of Big Five traits and social desirability did not influence the experimental effects. Furthermore, very weak or non-significant links were found between conspiracy theory related variables and individual differences and these were not related to CT belief change. There might be several reasons for these results: it is possible that the effect of individual differences on CT beliefs is smaller among Hungarians than in other countries. It is also possible that we could not measure any effect of individual differences on the acceptance of CTs with the specific CT material used in the experiment. Finally, it is also possible that other, longer or more sophisticated personality or social desirability measures would better demonstrate the possible role of individual differences. Contrary to individual differences, situational variables influenced the belief in conspiracy theories (e.g., the perceived competence and intelligence of the person who argued against the CT, increased the effectiveness of the belief change).

The present study is not without limitations. The effect sizes were not large. However, measuring the effectiveness of different reasoning or convincing strategies is not easy. In the present study, the number of arguments was balanced, but the length of the audio recordings was different in the different conditions. Further, studies should balance the number of arguments, their length and pretest the effectiveness of each argument. Needless to say that it is a time consuming task. If we consider the present study as an intervention, it can first be said that this is not a wise one, as direct and confronting strategies were used to convince individuals regarding CT reduction. Second, this experiment did not have the very solid theoretical background that a good intervention requires. Third, this study only measured the short-term effects of different CT reduction strategies. Fourth, it targeted a general population instead of a specific subgroup of individuals. Fifth, the timing of the experiment was not related to a big CT-related scandal, which could have influenced the effectiveness of the conditions.

Besides these numerous deficiencies, the present study shows that rational arguments can reduce CT beliefs, while ridiculing also appears to be somewhat effective. Future studies are needed in order to explore the boundaries of these results. But, after careful investigation of these conditions (culture, timing, different groups with different characteristics, different speakers, etc.), media campaigns can be designed and in collaboration with competent public speakers, different CT reduction strategies can be tested.

## Conclusion

Despite the extensive knowledge about the harmful effects of having CT beliefs, the reduction of CT beliefs with experimental methods is a relatively neglected topic of scientific investigation. In the present study, three convincing strategies were tested in order to reduce CT beliefs: rational arguments, ridiculing of CT believers, and expressing empathy toward the objects of CT beliefs. Providing rational arguments was found as being an effective strategy, along with providing ridiculing arguments, which could also reduce CT beliefs. Only very weak, or even non-significant links were found between conspiracy theory-related variables and individual differences. Considering these results and previous studies focusing on the benevolent effects of analytic thinking in CT belief reduction, it can be assumed that uncovering arguments regarding the logical inconsistencies of CT beliefs can be an effective way to discredit them. Our findings on the efficiency of rational argumentation go against the mainstream of the communication literature and “common wisdom,” as well as the current affective wave of social psychology emphasizing that emotions constitute the most important factor behind shaping beliefs and attitudes. Considering the modest effect sizes, we assume that rationality has a bigger impact on shaping (sometimes irrational) beliefs than previously expected, given that in the current communication environment, people are overloaded with emotional messages coming from ads, political and social campaigns. Future studies should also investigate the role of rationality and the “rationality heuristic” in belief change.

## Author contributions

GO contributed to the study design, manuscript writing, and data analyses; PK contributed to the study design, data gathering and interpretation, and literature review; BP contributed to the literature review and interpretation of the results and writing the manuscript; IT and BB contributed to the data analysis and interpretation, writing the manuscript, and the literature review; CR contributed to the manuscript writing and literature review. All authors commented on the draft and contributed to the final version, approved the publication of the manuscript, and agreed to be accountable for all aspects of the work.

### Conflict of interest statement

The authors declare that the research was conducted in the absence of any commercial or financial relationships that could be construed as a potential conflict of interest.

## Footnotes

^1^According to the theory of planned behavior, a behavior occurs more probably if (a) the individual has a positive attitude toward, (b) has a sense of being able to control the given behavior, and (c) the subjective norms support this particular behavior. This combination of attitudes, beliefs, and subjective norms leads to increased behavioral intention, which in turn results in higher probability of the actual performance.
