# Supplementary material for: Changing Conspiracy Beliefs through Rationality and Ridiculing
Source: Front Psychol. 2016 Oct 13;7:1525. doi: 10.3389/fpsyg.2016.01525 (PMC5061726; doi:10.3389/fpsyg.2016.01525)
Supplement: Supplementary file 2 [file DataSheet2.docx]

**Appendix 1**

**The operation of the world economy**

In the second half of the 19th century, the frequently recurring crises were induced by the hidden international financial cartel and the interest-group that controlled the finances from the shadows. Hungary, cornered into the trap of debt, became the province of the only superpower, the global financial empire. The financial technocrats of the one-party state, the current servants of the global financial empire, set up and operate the financial pumps for the global elite. As a result, the Hungarian national revenue is flowing out of the country.

One of the main reasons behind the deficit of the Hungarian budget and the external trade is that the EU is continuously sacking Hungary instead of supporting it. The EU is the institute of the organized private power for the cheap and voluntary elimination of the nation-states standing in the way of the super-bankers. The EU became a redundant and over-bureaucratized water-head. The advantages—the freer movement of manpower and products, the reduction of duty, closer cooperation among the states of Europe—could have been achieved more cheaply and more effectively without it. We should not have given up our national sovereignty, autonomy, the separate Hungarian jurisdiction. The EU is unnatural, because it is not a community like a nation or a family which is essential for the biological and societal reproduction. These two families are the necessary requisites of life. However, the EU is a parasitic formation without any function.

Upon the collapse of the monetary system, the speculators, who provoked the crisis, asked the state to save the banking system, because if the normal inter-bank lending is not restored, they cannot finance the real economy: the manufacturing plants will simultaneously shut down, become bankrupt, and millions of people will end up on the streets. At that time, the government put its hand in the pocket of the taxpayers and charged them with the saving of the plunging bankers by using public money. We still cannot know why the plunging bankers were preferred as opposed to the value-producing real economy.

The international financial community, which constitutes the central core of this global system, includes the super-wealthy money dynasties in the City of London. Belonging to this “global money cartel” or monetary group referred to as “Empire of the City of London” are the members of the House of Rothschilds, the House of Warburgs, the House of Barings, in addition to the Banking House of Brown-Shipley, the House of Schroeders, the House of Morgan-Grenfels, and the Banking House of Lazard Freres. The listed, investing banker houses have their sub-offices in New York, Paris, Hamburg, Switzerland, and Hong-Kong. They provide the monetary tools for the global elite to sustain the politics of power balance. The organized system of this global elite includes the masonic schools, the different branches of the Illumati, the Cabalists and the Sufis. It also includes the institutions of the globally built corporate mass communication, the secret services that are not just rival of each other, but cooperate as well, the intelligence agencies, and the financing and execution of the organized secret programs. These secret programs include the destabilization of countries and governments that slipped out of control, and the system change in the case of the disobedient governments. It also includes the supervision of the drug-traffic which allows the hidden financing of programs that cannot be supported by the official national budget.

The Zionist world strategy, in concreto, can be seen as the global strategy to achieve world hegemony. It can be successfully accomplished if humanity does learn anything about it. For this reason, Zionists practically issued war against everyone who knew about their activity, analyzes it, judges it, and understands and publishes about their world strategy. The international Jewry created the bolshevism, and the same power disintegrated the Soviet Union. Today, the United States is the next. The Zionist monetary world elite have already successfully gained control over the political system of the United States.

Only with the initiation of the public money system—that restores the sovereignty of the people—can we avoid the current morbid and decadent civilization of usury and convert to a healthier, person-centered society in Hungary as well. (…) The explosion can be avoided globally and within a country as well, if we restore the public money system and expel the division of the money cartel that is made up of corporate bodies trained for cheating and looting.

**Appendix 2.**

**Rational condition**

The text is not logical and consistent on multiple occasions. First of all, the framing regarding the European Union is factually wrong: Hungary is one of the main beneficiaries of the EU membership. In 2013, the EU balance of Hungary was 4956.9 million euros that is Hungary received much more funds from the EU than the amount it deposited. After Poland and Greece, Hungary had the best deal nominally. When the balance is measured in proportion to the Gross National Income (GNI), the support of our country is even more outstanding. In 2013, the balance added up to 5.33% of the GNI; it is the highest among the 28 member states. If we logically think through that the EU is giving almost 5 billion euros each year to Hungary for development, it is hard to conceive of this as the “cheap and voluntary” elimination of a nation-state.

No direct or indirect proof supports that a secret society is controlling the world or Hungary. The most important political players of the world are those world-powers whose operations are public (for example, the United States of America). The economic power is transparent as well, the world’s largest banks, enterprises are present on the stock market, their operations are overseen, their accounting is public, and anybody can acquire public information about their management. The invisible players behind the scenes cannot even exert their power. To have influence, it is necessary to have publicity, the world’s most important political and economic players are right before our eyes, we just have to notice them.

Contrary to the text, the world’s financial core does not consist of the above-mentioned housed, nor the Zionist/Israeli/Jewish lobby. The British banks are not the most important. The Chinese banks dominate the international ranking of the banks based on the value of their tools. Among the top 10 biggest banks, 4 are Chinese, while in the top 50, 10 are Chinese, 6 are American, and 5-5 are Japanese, French, and British. In the top 10 enterprises, ranked by Forbes, 5 are Chinese (among them the Chinese ISBC with a wealth of 3124 billion dollars) and 5 are American (among them the JP Morgan with 2353 billion dollars).

The “Zionist lobby” mentioned by the text actually exists, but its operation is also open and transparent. There are organizations in the United States that want to influence governmental decision making, like the Simon Wiesenthal Center and the American Jewish Committee. They often hold public assemblies where they also discuss in what they successfully influenced the government. And many other groups have similar public organizations in the United States: Muslims, Armenians, etc.

If the banks were not supported by the states, then everybody would be without money now. What the text formulates as a desire, the removal of the banks, its logical consequence would be the monetary and economic collapse and multiple millions of people would be without money, credits, savings, and jobs. Besides, the Hungarian state collected much more money from the banks in total since the crisis than it gave them in support after the beginning of the crisis. For instance, in 2008, they gave 600 billion Hungarians forints (HUF) to the banks in support, while they collected 1222 billion HUFs from them in the form of bank tax alone. Moreover, with the final repayment of 2012, the banks lost 260 billion, in addition to the other 1000 billion HUFs that was collected from the banks during their final accountability. In sum, between 2008 and 2015, the state gave 600 billion support and took 2842 billion HUFs from the banks.

**Ridiculing condition**

The fight against the “global financial empire” and other invisible enemies is the hobby and craze of conspiracy theory believers. The important thing is to always have an evil with whom people can be scared, like the bogeyman crawling out from under the children’s bed. It is the best to choose a suspicious group with a bad reputation as enemy: secret societies, the House of Rotschilds, the illuminati, the Cabalists, international financial capital, Jews, etc. There are people who, following this train of thought, think that Lizard people want to take control over us, and for example the American presidents are disguised Lizard people in reality. Believable, right? The Facebook page of the most famous Lizard people believer, David Icke, is followed by half a million people. According to a research, 2% of the Americans, more than 6 million people, believe in the Lizard people theory. Obviously, it is easier to scare with a bogeyman than to think logically. It has also been proven by research that logical thinking is not a strength of the conspiracy believers. According to a British study, people believing Osama Bin Landen to still be alive, despite the official version, also believe in that he was already dead when American soldiers found him. He lives and dies at the same time. Believable, right?

The creators of conspiracy theories have excellent recipes: always be suspicious and skeptical, it will make you smarter. Use a lot of foreign words; it will make you even smarter. Use a lot of concepts whose meaning is not clear to anyone, not even you, and express yourself strongly with these ambiguous concepts. Besides looking super intelligent, nobody can refute what you say. Why? Because what is said is completely beyond reason. Irrationality cannot be refuted. Can you, for example, refute the baloney of a small child? Exactly, you cannot. Let’s have a look at the following sentence from the text: *“The financial technocrats of the one-party state nomenclature, the current servants of the global financial empire, set up and operate the financial pumps for the global elite.”*. I will offer the Nobel Prize for those who can discover the seed of meaning in the sentence. Who could the financial technocrats of the one-party state nomenclature be? What the hell can a money empire be? But it is sure that bankers took all the public moneys. There is no alternative explanation for this. And how does a monetary pump look exactly? The whole should be imagined as a banker sitting on the monetary pumps, pumping the money out of people’s pockets? Wait a second here, whose money is in the bank? Yeah, everybody’s. Let’s expel the banks, but then we will expel our own money.

Nevertheless, many can be persuaded with texts like these, one can gain fans, one can pick up girls or boys. Try it yourself! Talk about secret conspiracies running in the background with foreign, ambiguous words, with dubious characters and in between repeat multiple times that “*we still do not know today…*”; “*it cannot be a coincidence, right?*”; and ask the question: “*whose interest is all this?*” “*Guess who?*”. The success is guaranteed, people will hummingly think that you are smart. For those who agitate you, say another three, undefined, six-syllable-long concept in a ten-line-long sentence, then raise your eyebrow and look at the protester interrogatively. You will be the star of the party, guaranteed! If you are doing it well enough, you can even make a living out of it.

**Empathic condition**

The different influential societies, families, bankers are often accused that they maliciously control countries, the EU and the events of the world from behind the scenes. As a consequence, Hungary often appears as a marionette that is controlled by them. Along other groups in the text, the global monetary empire, the super power whose leaders are the secretly controlling Zionists, alias Jews. It is important to know that the same scapegoat theory has already led to tragedy in multiple occasions. Similar theories constituted the Nazi propaganda about the world dominance of the Jews and resulted in the killing of 6 million people, women and children among them. Not only were the Jews the target of these conspiracy theories by any means. In the Middle Ages, the Jews were accused of poisoning water wells and murdering virgin girls. In the early eras of Christianity, in Ancient Rome, the same accusations were made against the Christians, and they were hunted and killed based on these accusations. A similar logic is behind current Christian prosecutions. The influential societies are often vested with demonic power so that they can be stripped of their goods. For example, the Knights Templar, who played an important role in the Crusades and who were loyal to the Christian values, were accused in the 13. century of making a pact with the Devil – in reality, people were just jealous of their success and wealth. The wealth of the Templars was confiscated, its members were tortured and burnt. It was later revealed that they were framed. The logic that there is a conspiracy behind everything could easily turn anyone into mortal enemy. Conspiracy theories are dangerous: you can also be a conspirator. Do you know the feeling when you are disadvantaged, because you are a woman, you belong to a minority or you are different from the majority for a reason? Multiple your experiences by one thousand and then you can an approximate the feeling of being accused of conspiracies.

The banker families mentioned in the text indeed dispose over considerable wealth. But this does not mean that they think in a cold, calculating, ruthless and rational manner that is supposed by the theory above: they control the whole European Union, and they pick the money out of the taxpayers’ pocket through the nation states. The members of these families are sensitive humans with their own problems as much as anyone else. They make good and bad decisions in their work-life and private life just like anyone else. They love, hate, and suffer as much as anyone else. For example, Kate Rotschild, who is referred to as the “black sheep of the Rotschild family”, divorced from her blue-blooded husband to marry a rapper, she started a record company, and made a big scandal when she appeared in social events in tracksuits.

**Control condition**

If we examine the capriciousness and unpredictability of the weather relying on human memory, than even more could think that “something is not right” with our everyday weather. In reality, however, there was no exceptional change. On our geographic latitude, the capriciousness of the weather was always typical. This was the case centuries ago, written records from this latitude talk about it. It happened that there were rainier, drier, hotter and colder periods than average, during both summer and winter. We can cite the chronicler who wrote the following about the Hungarian winter of 1695-96: “Somebody swore that time have changed. Winter became summer so much that there is nothing wintery about winter and there was no cold, no freeze, no snow during the otherwise coldest months. Since we could remember, this winter was the mildest.” If we jump forward in time, the winter of 1989-90 was unusually mild in our homeland. There was measureable snow-blanket only for 1-2 days altogether. And the examples can be listed pro and contra. Maybe we feel that our weather is more capricious and unpredictable nowadays, because we hear about the weather events through the media. The weather trend defines how we spend our leisure time, which by the way is less and less. 200 years ago, for example, if it snowed less in the Alps, then it did not jeopardize the programs of tens of thousands of skiers. Then of course our opinions are influenced by the “*buts*” as well: “*But in my youth, seasons orderly followed each other. During winter, there was winter, during summer, summer.*” There is truth in it, and there is not. But we just remember the “good things”, that it is all right.

The weather of the Carpathian Basin is greatly influenced by the dominating wind. If the meteorological conditions are given, in 24 hours, 20-30 centimeters of snow can fall from the clouds of Mediterranean cyclone coming from the South-Southwest. However, there are winters when no Mediterranean cyclone is formed. The strong Northeastern, Eastern stream floats dry, freezing Siberian air above us. It is still a mystery for the meteorologists to know what streams will be final in a given time and area. For this reason, it is not possible to create reliable, seasonal forecasts.

Nowadays, we can only have forecast with a reliable tendency up to a maximum of two weeks. This means that for these two weeks, it can be forecasted whether the weather will be dry, cold, or mild. In the long run, it is very hard to “predict”. Forecasts with six months in advance can be made, but these are very unreliable. We share the valid, long-term forecast of this winter with the readers. According to this, the December of 2005 in the Carpathian Basin has average temperature and it is rainier than the average (!), the January of 2006 is colder and rainier than the average (!), February is a little colder than average, and it entices with an average amount of rainfall. We will see. Unfortunately, the vernacular weather regulations do not help in making the long-term forecasts more predictable.
